# Effects of acid-sensing ion channel-1A (ASIC1A) on cocaine-induced synaptic adaptations

**DOI:** 10.3389/fphys.2023.1191275

**Published:** 2023-06-14

**Authors:** Subhash C. Gupta, Rebecca J. Taugher-Hebl, Jason B. Hardie, Rong Fan, Ryan T. LaLumiere, John A. Wemmie

**Affiliations:** ^1^ Department of Psychiatry, University of Iowa, Iowa City, IA, United States; ^2^ Department of Veterans Affairs Medical Center, Iowa City, IA, United States; ^3^ Department of Psychological and Brain Sciences, University of Iowa, Iowa City, IA, United States; ^4^ Iowa Neuroscience Institute, University of Iowa, Iowa City, IA, United States; ^5^ Interdisciplinary Graduate Program in Neuroscience, University of Iowa, Iowa City, IA, United States; ^6^ Department of Molecular Physiology and Biophysics, University of Iowa, Iowa City, IA, United States; ^7^ Medical Scientist Training Program, University of Iowa, Iowa City, IA, United States; ^8^ Department of Neurosurgery, University of Iowa, Iowa City, IA, United States

**Keywords:** cocaine, synaptic plasticity, ASIC1A, structural plasticity, nucleus accumben

## Abstract

Chronic drug abuse is thought to induce synaptic changes in nucleus accumbens medium spiny neurons (MSNs) that promote subsequent craving and drug-seeking behavior. Accumulating data suggest acid-sensing ion channels (ASICs) may play a critical role. In drug naïve mice, disrupting the ASIC1A subunit produced a variety of synaptic changes reminiscent of wild-type mice following cocaine withdrawal, including increased AMPAR/NMDAR ratio, increased AMPAR rectification, and increased dendrite spine density. Importantly, these changes in *Asic1a* ^*−/−*^ mice were normalized by a single dose of cocaine. Here we sought to understand the temporal effects of cocaine exposure in *Asic1a* ^*−/−*^ mice and the cellular site of ASIC1A action. Six hours after cocaine exposure, there was no effect. However, 15 h, 24 h and 4 days after cocaine exposure there was a significant reduction in AMPAR/NMDAR ratio in *Asic1a* ^*−/−*^ mice. Within 7 days the AMPAR/NMDAR ratio had returned to baseline levels. Cocaine-evoked changes in AMPAR rectification and dendritic spine density followed a similar time course with significant reductions in rectification and dendritic spines 24 h after cocaine exposure in *Asic1a* ^*−/−*^ mice. To test the cellular site of ASIC1A action on these responses, we disrupted ASIC1A specifically in a subpopulation of MSNs. We found that effects of ASIC1A disruption were cell autonomous and restricted to neurons in which the channels are disrupted. We further tested whether ASIC1A disruption differentially affects MSNs subtypes and found AMPAR/NMDAR ratio was elevated in dopamine receptor 1-expressing MSNs, suggesting a preferential effect for these cells. Finally, we tested if protein synthesis was involved in synaptic adaptations that occurred after ASIC1A disruption, and found the protein synthesis inhibitor anisomycin normalized AMPAR-rectification and AMPAR/NMDAR ratio in drug-naïve *Asic1a* ^*−/−*^ mice to control levels, observed in wild-type mice. Together, these results provide valuable mechanistic insight into the effects of ASICs on synaptic plasticity and drug-induced effects and raise the possibility that ASIC1A might be therapeutically manipulated to oppose drug-induced synaptic changes and behavior.

## Introduction

Drug abuse induces synaptic adaptation in the reward circuit, which are thought to underlie drug craving and relapse, and contribute to the development of substance use disorders (Nestler, 2001; Luscher, 2016; Wolf, 2016). The nucleus accumbens (NAc) is a vital brain area of the reward circuit comprised of heterogenous populations of D1 and D2 medium spiny neurons (MSNs) and a location where drug-induced adaptations have been observed (Gerfen, Engber et al., 1990; Kalivas, 2009; Wolf, 2010; Kim, Park et al., 2011; Lobo and Nestler, 2011). NAc core (NAcc)-MSNs undergo a host of drug-induced adaptations, such as an increase in the AMPAR/NMDAR ratio, recruitment of GluA2-lacking calcium-permeable AMPA receptors (CP-AMPARs) due to heightened GluA1 protein synthesis, and changes in dendritic spine density (McCutcheon, Wang et al., 2011a; Purgianto, Scheyer et al., 2013; Loweth, Tseng et al., 2014a; Scheyer, Wolf et al., 2014; Christian, Wang et al., 2017; Stefanik, Milovanovic et al., 2018; Werner, Stefanik et al., 2018). The cumulative effects of these neuroadaptations are thought to promote drug-seeking behaviors and addiction (Cornish and Kalivas, 2000; Wolf, 2016).

Previously, we found that acid-sensing ion channel-1A (ASIC1A) in the NAcc participates in synaptic transmission and influences synaptic responses to drugs of abuse, such as cocaine (Kreple, Lu et al., 2014). ASICs are homo- and hetero-trimeric cation channels consisting of ASIC1A, ASIC2A, and ASIC2B subunits and are activated by extracellular acidosis (Wemmie, Taugher et al., 2013). The ASIC1A subunit is required for activation by pH changes within the physiological range (from pH 7.4 to 5.0) (Wemmie, Chen et al., 2002; Askwith, Wemmie et al., 2004). Transient extracellular acidification evokes ASIC-mediated currents, which are attenuated by ASIC inhibitors or ASIC1A disruption (Wemmie, Chen et al., 2002; Kreple, Lu et al., 2014; Gupta, Ghobbeh et al., 2022).

ASIC1A is present in synaptosomal brain fractions and is located in postsynaptic dendritic spines (Zha, Wemmie et al., 2006; Gupta, Ghobbeh et al., 2022), where it is well-positioned to detect changes in extracellular pH. One potential source of acidosis that may activate these channels is presynaptic vesicles, which are highly acidic (pH 5.5) and release protons into the synaptic cleft during neurotransmission (Miesenböck, De Angelis et al., 1998; Du, Reznikov et al., 2014; González-Inchauspe, Urbano et al., 2017). Supporting this possibility, in the presence of AMPAR, NMDAR, and GABA_A_R inhibitors, a component of the EPSC remains that depends on ASIC1A and is blocked by ASIC inhibitors (Du, Reznikov et al., 2014; Kreple, Lu et al., 2014; González-Inchauspe, Urbano et al., 2017; Gupta, Ghobbeh et al., 2022).

ASIC1A-dependent EPSCs may contribute to synaptic stability, as disrupting ASIC1A produces functional and structural changes in NAcc MSNs, including increased in AMPAR/NMDAR ratio, increased CP-AMPARs and increased density of dendritic spines (Kreple, Lu et al., 2014). These rearrangements resembled adaptations in NAcc MSNs observed following cocaine withdrawal and were similarly sensitive to the effects of a single dose of cocaine (Kreple, Lu et al., 2014; Gupta, Ghobbeh et al., 2022). At the behavioral level, ASIC1A disruption increased cocaine-reinforced behaviors, including cocaine conditioned place preference (CPP) (Kreple, Lu et al., 2014) and cocaine-evoked locomotor responses after withdrawal (Jiang, Wang et al., 2013). Moreover, disrupting ASIC1A specifically in the NAcc increased cocaine CPP, while restoring ASIC1A expression to the NAcc in *Asic1a* ^*−/−*^ mice normalized it, implicating the NAcc as a key site of ASIC1A action (Kreple, Lu et al., 2014).

With this background, the present study investigated the role of ASIC1A disruption and the temporal effects of acute cocaine exposure on synaptic physiology. It also investigated the effects of acute cocaine exposure on dendrite spine morphology in *Asic1a* ^*−/−*^ mice. In addition, it explored whether the effects of ASIC1A disruption on NAcc MSNs are autonomous to the cells expressing ASIC1A, or whether they might be indirect. Finally, it studied the role of ASIC1A in different MSNs subtypes and the involvement of protein synthesis in ASIC1A-dependent synaptic adaptations.

## Materials and methods

### Mice

C57BL/6J mice matched for age (8–12 weeks) and sex were used. Mice were held on a standard 12-h light-dark cycle, contained in groups of 2–5 littermates, and fed standard chow and water *ad libitum*. Experiments were carried out during the light cycle. *Asic1a* ^*+/+*^, and *Drd1a-tdTomato* mice (stock #016204) were obtained from Jackson Laboratory. *Asic1a* ^*−/−*^ and *Asic1a* ^*loxP/loxP*^ mice were generated as previously described (Wemmie, Chen et al., 2002; Kreple, Lu et al., 2014). *Asic1a* ^*+/+*^:*Drd1a-tdTomato* and *Asic1a* ^*−/−*^:*Drd1a-tdTomato* were generated crossing *Drd1a-tdTomato* mice with *Asic1a* ^*+/−*^ mice. The University of Iowa Animal Care and Use Committee approved all experiments and animal care followed the National Institutes of Health standards.

### Slice preparation and electrophysiology

Acute slices were obtained and electrophysiological recordings were performed as described previously (Kreple, Lu et al., 2014; Gupta, Ghobbeh et al., 2022). AMPAR/NMDAR ratio and AMPAR-rectification were measured as previously described (Gupta, Ghobbeh et al., 2022). Briefly, AMPAR/NMDAR ratio of evoked-EPSCs were calculated from AMPAR-EPSC amplitude at −70 mV by the NMDAR-EPSC amplitude at +50 mV, using the late component of the NMDAR EPSC, 60 ms after the onset. An evoked AMPAR-mediated EPSCs were measured at membrane potentials of −70, −50, −30, −10, +10, +30, and +50 mV and current-voltage (I–V) relationship of AMPAR EPSCs were plotted. Rectification index of AMPAR-mediated EPSCs was calculated as ratio of I_-70 mV_ divided by I_+50 mV_. NASPM sensitivity of AMPAR EPSC was evaluated by collecting 20 to 25 baseline sweeps in ACSF and 15 min following NASPM application (200 μM, Alomone Lab) while holding the cell’s voltage at −70 mV. To study the role protein synthesis on these measures, acute brain slices were pre-treated with 25 µM anisomycin (Sigma-Aldrich) for 1 h.

### Cocaine injection

To test the effects of acute cocaine, a single dose of cocaine (10 mg/kg, i.p.) or saline (0.9%, i.p.) was injected in the homecage, and electrophysiology was performed 6, 24 h and 7 days post injection. DiI labeling for dendritic spine study was performed 24 h post injection. Cocaine was kindly provided by the National Institute on Drug Abuse.

### Virus vectors

Adeno-associated viruses (AAV) expressing Cre recombinase or eGFP under control of a CMV promoter were injected into the NAcc as described previously (Kreple, Lu et al., 2014; Gupta, Ghobbeh et al., 2022). The Cre-injected group used a 70/30 mixture of AAV-CMV-Cre and AAV-CMV-eGFP to facilitate identification of virus-transduced neurons. Electrophysiology was performed at least 3 weeks after injection to allow for virus transduction.

### DiI labeling, dendritic spine imaging and analysis

Mice were perfused, tissue was harvested and NAcc MSN neurons (not D1R^+^ or D2R^+^-specific) were stained with DiI as described previously (Gupta, Ghobbeh et al., 2022). Dendritic segments were imaged with a confocal microscope (Zeiss 710), and spine numbers and morphology were characterized (Neuron Studio) as previously described (Gupta, Ghobbeh et al., 2022). Experimental groups were comprised of 3-4 animals per group, 3-4 neurons per animal, and 2-4 dendritic segments per neuron.

### Statistical analysis

Student’s *t*-test was used to assess statistical significance for experiments involving two groups. One-way ANOVA was used to determine the statistical significance of time dependent effect of cocaine. Two-way ANOVA was used to assess statistical significance for experiments involving more than two groups. Within the context of the full ANOVA planned contrast testing was used to test *a priori* hypothesized relationships between groups. ROUT test with Q = 1% was used to screen for outliers. Because normality tests are underpowered with samples of this size, distributions were assumed normal. *p* values less than 0.05 were considered significant. All bar graphs express values as mean ± s.e.m. All statistical analyses were performed using GraphPad Prism.

## Results

### Temporal dynamics of acute cocaine-evoked synaptic rearrangement in *Asic1a* ^*−/−*^ mice

We have previously demonstrated that acid-evoked current, and ASIC-mediated EPSCs were disrupted, and ASIC1A protein band was absent in the *Asic1a* ^*−/−*^ mice (Kreple, Lu et al., 2014). We further found that ASIC1A disruption elevated the AMPAR/NMDAR ratio at synapses in NAcc MSNs above levels typically observed in wildtype mice. Moreover, in *Asic1a* ^*−/−*^ mice, a single cocaine injection (10 mg/kg, i.p.) normalized this ratio toward wild-type levels when tested 24 h post-injection. By contrast, the same cocaine injection did not affect AMPAR/NMDAR ratio in wild-type mice (Kreple, Lu et al., 2014). These results suggested that ASIC1A disruption increased sensitivity to cocaine and might provide a unique window into how cocaine influences synapses.

**FIGURE 1:**
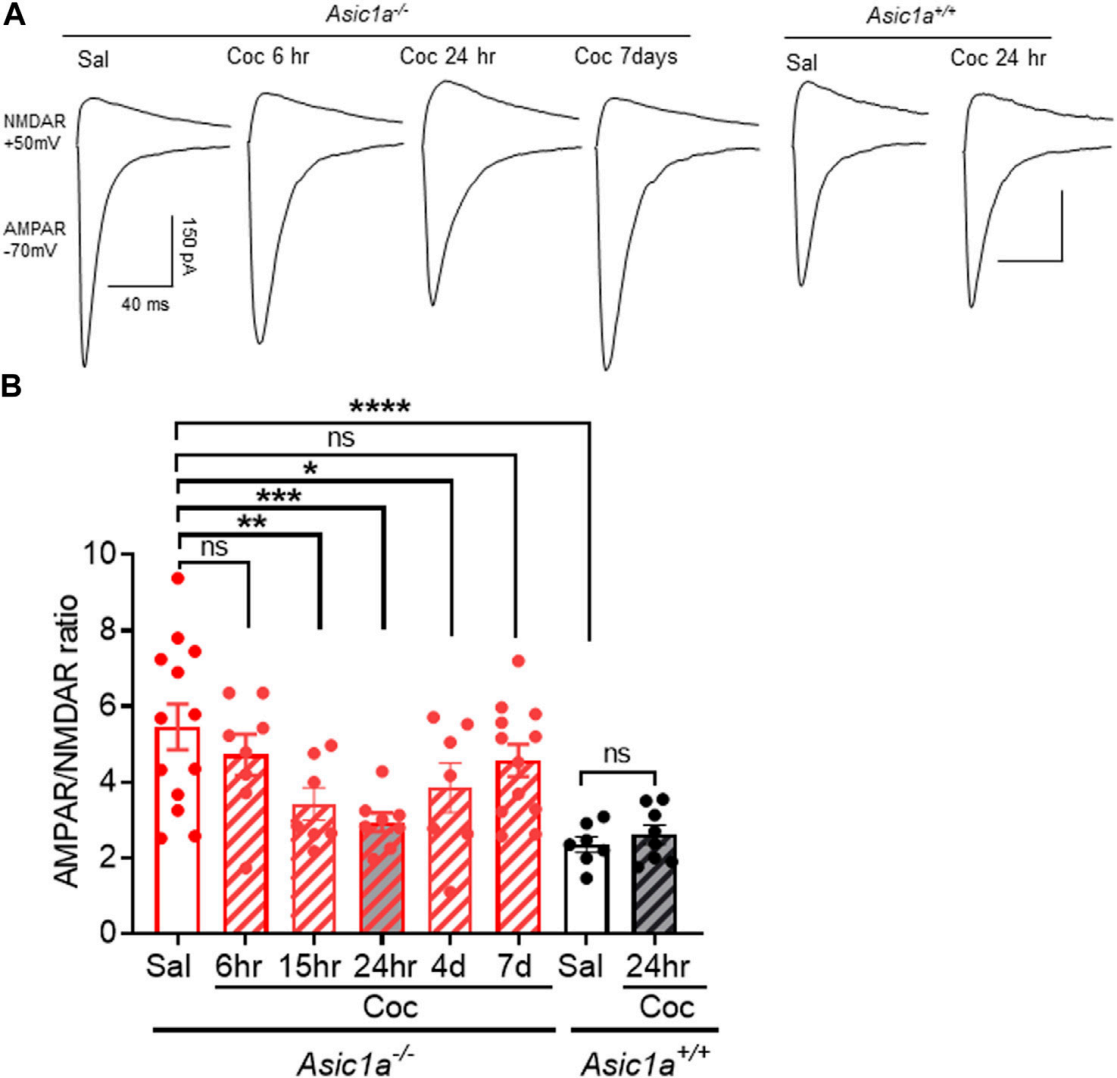
Acute cocaine exposure transiently altered AMPAR/NMDAR ratio in NAcc MSNs of *Asic1a* ^*−/−*^ mice **(A)** Representative traces of the AMPAR-mediated EPSC at −70 mV and the NMDAR-mediated EPSC at +50 mV from *Asic1a* ^*−/−*^ and *Asic1a* ^*+/+*^ mice. **(B)** AMPAR/NMDAR ratio is significantly higher in saline-injected (Sal) Asic1a^−/−^ *compared to Asic1a* ^*+/+*^, ****p < 0.0001. AMPAR/NMDAR ratio was not changed at 6 h after a single cocaine injection in *Asic1a* ^*−/−*^ Sal vs. Coc (6 h), p = 0.2715. However, AMPAR/NMDAR ratio in *Asic1a* ^*−/−*^ mice was reduced at 15 h (Coc vs. Sal **p = 0.0044), 24 h (Coc vs. Sal ***p = 0.0003) and 4 days (Coc vs. Sal *p = 0.0228) *n* = 7–13 neurons. The effect of cocaine was transient, and the AMPAR/NMDAR ratio returned to baseline levels by 7 days post cocaine injection (Sal vs. Coc (7 days) *p* = 0.1358). AMPAR/NMDAR ratio did not change in *Asic1a* ^*+/+*^ after a single cocaine injection [Coc (24 h) vs. Sal *p* = 0.734, *n* = 7—8 neurons]. One-way ANOVA, treatment [F(5.50) = 7.372, *p* < 0.0001].

In the present study, we sought to learn more about these mechanisms. An important and unknown characteristic of the unusual cocaine sensitivity of *Asic1a* ^*−/−*^ mice is its temporal dynamics. How quickly do these synaptic rearrangements occur after cocaine injection? And how long do they last? To help answer these questions, we injected a single dose of cocaine (10 mg/kg, i.p.) in *Asic1a* ^*−/−*^ and *Asic1a* ^*+/+*^ mice and quantified AMPAR/NMDAR ratio in NAcc MSNs at subsequent time points: 6, 15, 24 h, 4 and 7 days post-cocaine injection (Figures 1A, B). Interestingly, 6 h post-cocaine injection, we found that AMPAR/NMDAR ratio in *Asic1a* ^*−/−*^ mice was unchanged from baseline levels observed in saline-injected *Asic1a* ^*−/−*^ controls and elevated compared to saline-injected wildtype controls (Figure 1B). However, by 15 h post-cocaine injection until at least 4 days AMPAR/NMDAR ratio in *Asic1a* ^*−/−*^ mice was significantly reduced relative to saline-injected *Asic1a* ^*−/−*^ mice and approached the values observed in both saline-injected and cocaine-injected *Asic1a* ^*+/+*^ mice. By 7 days the AMPAR/NMDAR in *Asic1a* ^*−/−*^ mice had returned towards the baseline levels observed in saline-injected *Asic1a* ^*−/−*^ mice.

### Cocaine injection normalized AMPAR rectification in *Asic1a* ^*−/−*^ mice

Rectification of AMPAR-mediated synaptic currents provides a useful measure of AMPAR subunit composition (McCutcheon, Wang et al., 2011b; Purgianto, Scheyer et al., 2013; Kreple, Lu et al., 2014; Gupta, Ghobbeh et al., 2022). Synaptic recruitment of CP-AMPARs increases inward rectification (Conrad, Tseng et al., 2008; Mameli, Halbout et al., 2009; Ferrario, Loweth et al., 2011; Purgianto, Scheyer et al., 2013; Kreple, Lu et al., 2014; Loweth, Tseng et al., 2014; Gupta, Ghobbeh et al., 2022).

**FIGURE 2:**
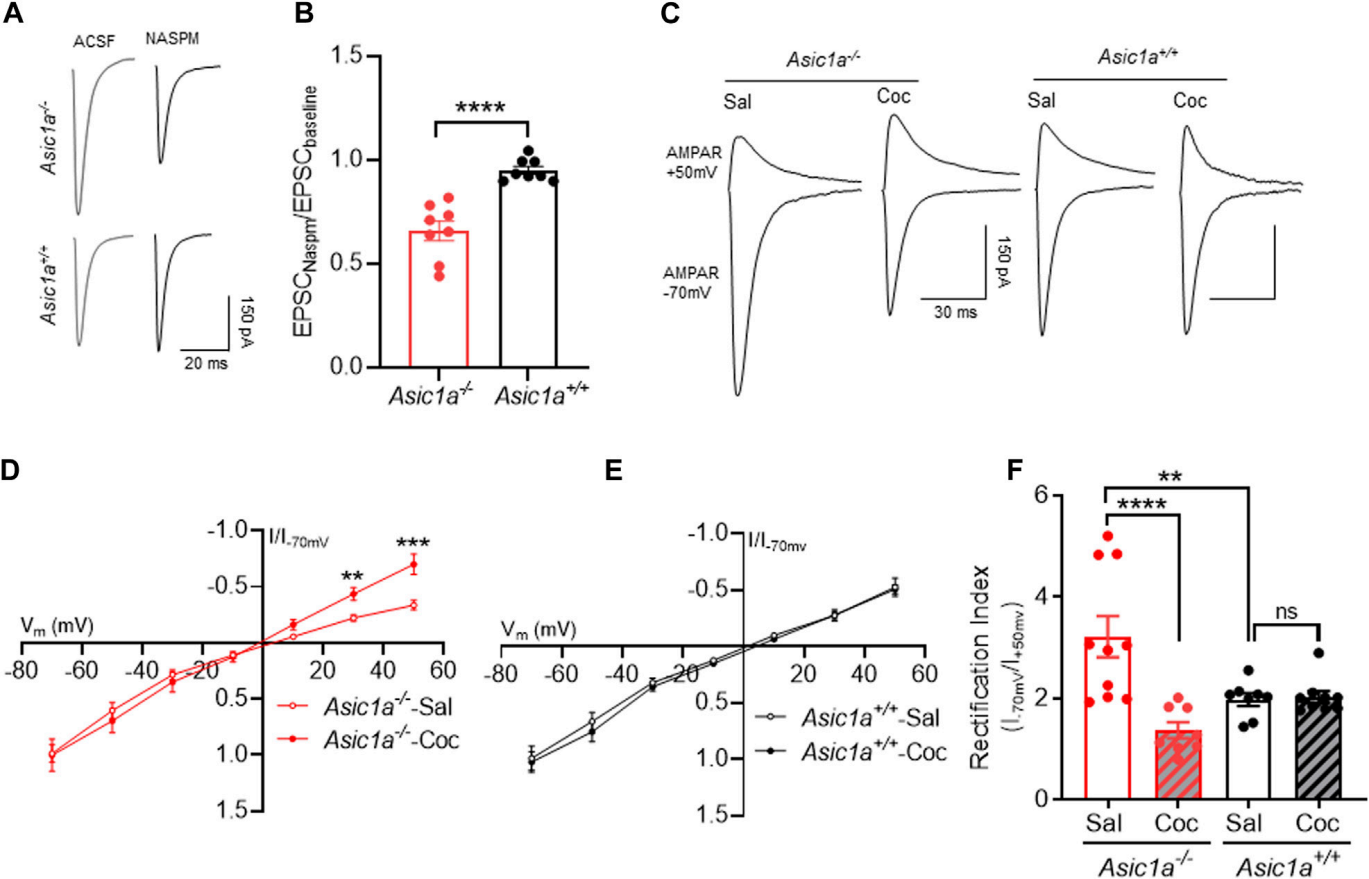
Cocaine treatment normalized AMPA receptor subunit composition in *Asic1a* ^*−/−*^ mice 24 h post injection **(A)** Representative trace of AMPAR-mediated evoked EPSCs at −70 mV in NAcc in presence of ACSF and NASPM **(B)** ASIC1A disruption increases NASPM sensitivity in NAcc of *Asic1a* ^*−/−*^ mice. *Asic1a* ^*−/−*^ vs. *Asic1a* ^*+/+*^, t (14) = 5.731, *p* < 0.0001, *n* = 8 neurons from 4 mice. **(C)** Traces of AMPAR-mediated EPSCs at −70 mV and + 50 mV in NAcc MSNs. **(D)** The I-V curve showed decrease in inward rectification in cocaine-treated *Asic1a* ^*−/−*^ *mice* compared to saline-treated *Asic1a* ^*−/−*^ (t (15) = 3.896 **p = 0.0014 (at 30 mV), t (15) = 4.218 ***p = 0.0007 (at 50 mV), *n* = 9 neurons from 4 mice and 11 neurons from 5 mice, respectively. **(E)** Cocaine treatment did not change inward rectification in I-V curve in the *Asic1a* ^*+/+*^ mice t (15) = 0.0216, *p* = 0.9821 (at 30 mV), t (15) = 0.222 *p* = 0.8273 (at 50 mV), *n* = 7 neurons from 4 mice and 6 neurons from 4 mice, respectively. **(F)** Loss of ASIC1A increases rectification index (RI) in saline-treated mice and cocaine treatment attenuates RI in Asic1a^−/−^ *but not in Asic1a* ^*+/+*^ mice [Interaction F (1, 31) = 13.36, *p* = 0.0009, two-way ANOVA. Planned contrast; Sal *Asic1a* ^*+/+*^ vs. Asic1a^−/−^ **p = 0.0018, Coc vs. Sal Asic1a^−/−^ ****p < 0.0001, Coc vs. Sal *Asic1a* ^*+/+*^ *p* = 0.892].

Therefore, we hypothesized that changes in AMPAR/NMDAR ratio observed above in *Asic1a* ^*−/−*^ mice may be due at least in part to altered recruitment of CP-AMPARs. To test this hypothesis, we quantified the sensitivity to the CP-AMPAR subunit blocker NASPM, and found NASPM sensitivity was increased in *Asic1a* ^*−/−*^ mice compared to *Asic1a* ^*+/+*^ mice (Figures 2A, B). Because CP-AMPARs also exhibit an increased rectification index, we also measured current-voltage (I-V) relationships of synaptic AMPAR responses in NAcc MSNs 24 h after injection of cocaine (10 mg/kg, i.p.) *versus* saline in *Asic1a* ^*−/−*^ and *Asic1a* ^*+/+*^ mice (Figures 2C–F).We found the rectification index (RI) was significantly elevated in saline-injected *Asic1a* ^*−/−*^ mice compared to their *Asic1a* ^*+/+*^ counterparts (Figure 2F), which is consistent with our previous observation (Kreple, Lu et al., 2014).

Moreover, 24 h after cocaine exposure, the elevated RI in the *Asic1a* ^*−/−*^ mice was reduced toward drug-naïve *Asic1a* ^*+/+*^ levels. The mean rectification trended even lower than wild-type levels, although the trend did not reach statistical significance. In contrast, cocaine exposure did not affect rectification in *Asic1a* ^*+/+*^ mice (Figures 2C–F).

These results suggest that, like AMPAR/NMDAR ratio, the RI in *Asic1a* ^*−/−*^ mice is similarly sensitive to acute cocaine exposure and within a similar timeframe. The results support the hypothesis that changes in the AMPAR/NMDAR ratio may be due, at least in part, to changes in AMPAR subunit composition.

### Acute cocaine exposure alters dendritic spines density and morphology in *Asic1a* ^*−/−*^ mice

Numerous studies have reported effects of cocaine exposure on dendritic spine density and morphology in a variety of brain areas (Spiga, Mulas et al., 2014; Edwards and Ersche, 2017).

Many of these studies tested effects of chronic cocaine exposure and/or withdrawal. Fewer studies have tested effects of a single cocaine injection. One study reported effects of a single cocaine injection (10 or 20 mg/kg, i.p.) on dendritic spine density and morphology in NAc shell (NAcsh) but not in NAcc MSNs. In that study, a single cocaine exposure increased dendritic spine density in the NAcsh, but not in NAcc 1h post-injection and remained high 1, 7, and 28 days after a single cocaine injection (Dos Santos, Salery et al., 2017).

We previously found that ASIC1A disruption increased dendritic spine density in NAcc MSNs, mainly due to increased thin and stubby spines (Kreple, Lu et al., 2014). However, in that study cocaine effects on spines were not tested. Since AMPAR/NMDAR ratio and RI in *Asic1a* ^*−/−*^ mice were acutely altered in response to acute cocaine exposure, we hypothesized dendritic spines number and morphology in *Asic1a* ^*−/−*^ mice would also be changed by cocaine.

**FIGURE 3:**
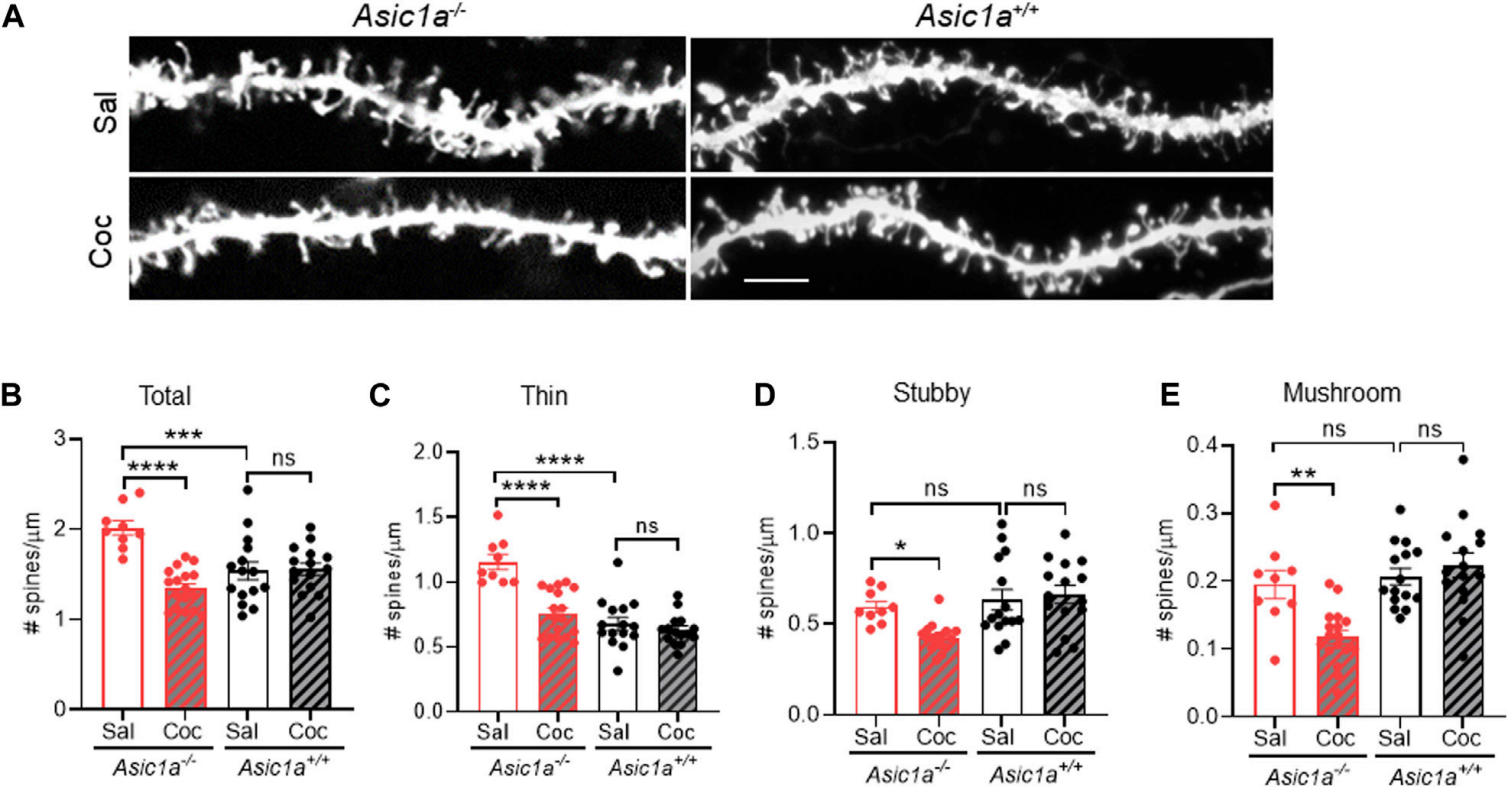
Acute cocaine treatment altered dendritic spine density and morphology in NAcc MSNs of *Asic1a* ^*−/−*^ mice **(A)** Representative images of dendritic spines in *Asic1a* ^*−/−*^ and *Asic1a* ^*+/+*^ mice after a single injection of cocaine (Coc) or saline (Sal). Scale bar 5 microns. **(B)** Acute cocaine exposure decreased total spine density in *Asic1a* ^*−/−*^ mice, not in *Asic1a* ^*+/+*^ mice [Interaction F (1, 52) = 19.38 *p* < 0.0001]. Planned contrasts: *Sal vs Coc Asic1a* ^*−/−*^ ****p < 0.0001 *n* = 9 neurons from 3 mice and 17 neurons from 4 mice, respectively; *Asic1a* ^*−/−*^ Sal vs. *Asic1a* ^*+/+*^ Sal ***p = 0.0002; Asic1a ^*+/+*^ Sal vs. *Asic1a* ^*+/+*^ Coc *p = 0.8964 n = 15 neurons from 4 mice*. **(C)** Thin spines were elevated after ASIC1A disruption. Acute cocaine exposure normalized thin spines [Interaction F (1, 52) = 14.55 *p* = 0.0.0004]. Planned contrasts: Sal vs. Coc *Asic1a* ^*−/−*^ ****p < 0.0001; *Asic1a* ^*−/−*^ Sal vs. *Asic1a* ^*+/+*^ Sal ****p < 0.0001; *Asic1a* ^*+/+*^ Sal vs. *Asic1a* ^*+/+*^ Coc *p* = 0.4475. **(D)** Stubby spines were reduced after cocaine treatment [Interaction F (1, 52) = 5.145, *p* = 0.0275]. Planned contrasts: Coc vs. Sal *Asic1a* ^*−/−*^ *p = 0.013; *Asic1a* ^*−/−*^ Sal vs. *Asic1a* ^*+/+*^ Sal *p* = 0.5741; *Asic1a* ^*+/+*^ Coc vs. *Asic1a* ^*+/+*^ Sal *p* = 0.6085. **(E)** Cocaine treatment attenuates mushroom spines [Interaction F (1, 52) = 9.771 *p* = 0.0029]. Planned contrasts: Coc vs. Sal *Asic1a* ^*−/−*^ **p = 0.0012; *Asic1a* ^*−/−*^ Sal vs. *Asic1a* ^*+/+*^ Sal *p* = 0.6154; *Asic1a* ^*+/+*^ Coc vs. *Asic1a* ^*+/+*^ Sal *p* = 0.4045.

To test this hypothesis, we compared the effects of a single dose of cocaine (10 mg/kg, i.p.) or saline in *Asic1a* ^*−/−*^ and *Asic1a* ^*+/+*^ mice. 24 h later we harvested the brain tissue and quantified dendritic spine density in NAcc MSNs. In saline-injected *Asic1a* ^*−/−*^ mice we found total dendritic spine density was increased compared to their wildtype counterparts (Figures 3A, B), which is consistent with previous findings (Kreple, Lu et al., 2014). However, surprisingly here we did not also observe an increase in stubby spines in *Asic1a* ^*−/−*^ mice (Figure 3D) as observed previously (Kreple, Lu et al., 2014). The reasons for this discrepancy are not clear but may be related to the different fixation methods, dyes, or neuron selection strategies employed in the two studies.

Supporting our hypothesis, cocaine exposure dramatically affected dendritic spine density in *Asic1a* ^*−/−*^ mice 24 h post-injection. Densities of total, thin, stubby, and mushroom spines were all significantly reduced compared to saline-injected *Asic1a* ^*−/−*^ mice (Figures 3A–E). These effects contrasted sharply with *Asic1a* ^*+/+*^ mice, where no effect of cocaine was observed on density of any spine type tested (Figures 3A–E) and this result is similar to Dos Santos et al., 2017. Together, these findings suggest that dendritic spines in *Asic1a* ^*−/−*^ mice, like AMPAR/NMDAR ratio and RI, are also sensitive to a single cocaine exposure 24 h post-injection. These observations suggest that ASIC1A disruption may destabilize spines, rendering them more labile to the effects of cocaine exposure.

### Cell autonomous effects of ASIC1A in NAcc MSNs

It is not yet known whether the effects of ASIC1A disruption on MSNs are specific to cells in which ASIC1A is disrupted or whether they might be secondary to the effects of ASIC1A elsewhere in the circuit. Because ASIC1A is robustly expressed in NAcc MSNs, we hypothesized that effects are specific to the cells in which the protein is disrupted (i.e., cell-autonomous).

**FIGURE 4:**
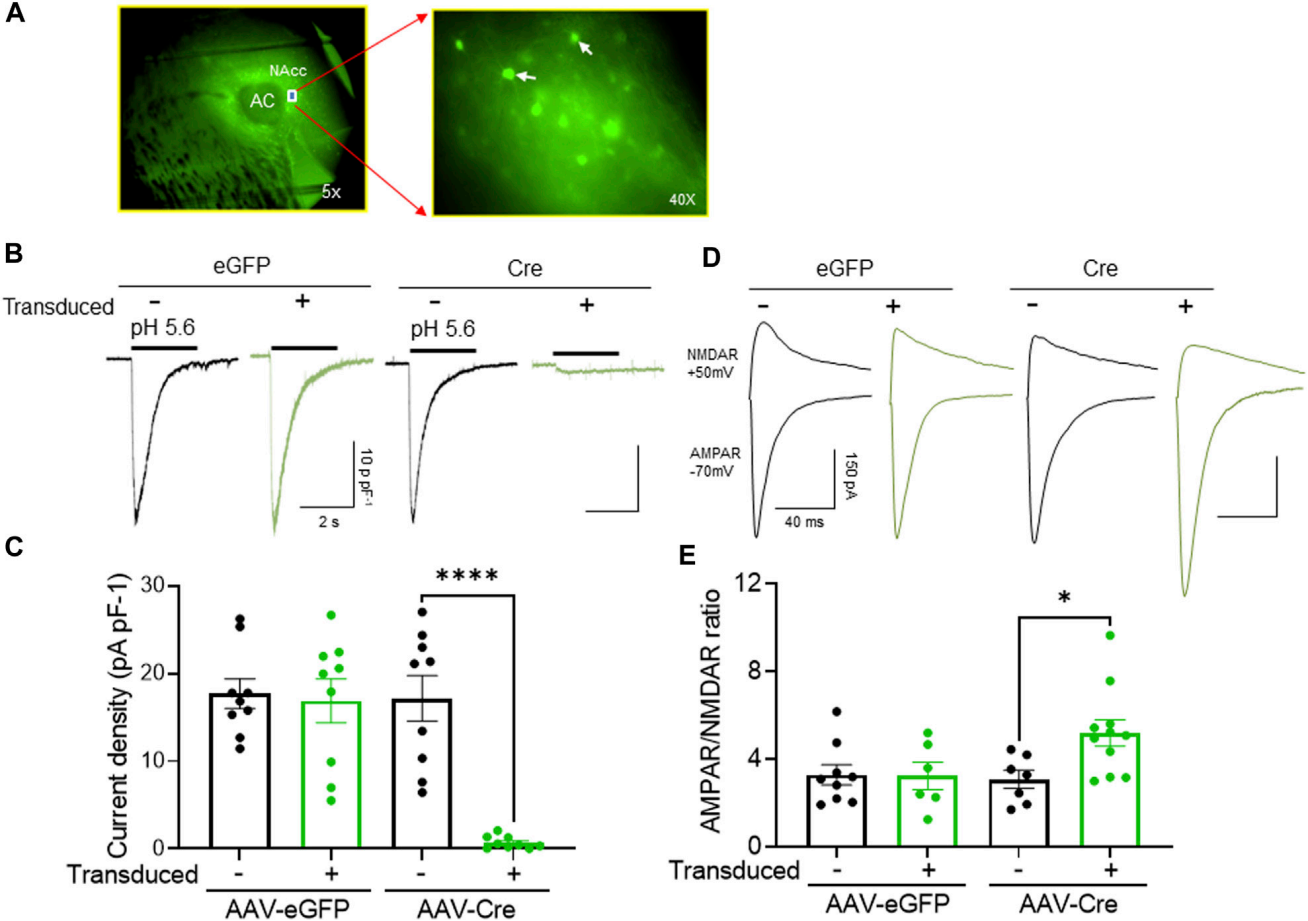
NAcc-specific ASIC1A disruption in *Asic1a* ^*loxP/loxP*^ mice inhibits ASIC-mediated current and increases the AMPAR/NMDAR ratio **(A)** Representative image from acute brain slices prepared for electrophysiological recording illustrating virus transduction in the NAcc MSNs under 5x (left) and 40x (right) with white arrow represents transduced MSNs. **(B)** Representative traces of acid-evoked current. **(C)** ASIC1A disruption in postsynaptic MSNs abolished acid evoked current [t (16) = 6.338 ****p < 0.0001, *n* = 9–11 neurons from 3 to 4 mice]. **(D)** Representative traces of AMPAR mediated EPSC and NMDAR mediated EPSCs **(E)** ASIC1A disruption in postsynaptic MSNs increases AMPAR/NMDAR ratio [t (16) = 2.574 *p = 0.0204, *n* = 9–11 neurons from 3 to 4 mice].

To test this hypothesis, we disrupted ASIC1A specifically in the NAcc core by injected a vector expressing Cre recombinase into *Asic1a* ^*loxP/loxP*^ mice (Figure 4A). As expected, the acid-evoked currents were almost eliminated in neurons transduced with Cre plus eGFP. Presumably, the small residual current observed was due to previously transcribed protein that had not yet been turned over. In contrast, acid-evoked currents were intact in neighboring non-transduced or in neurons transduced with the eGFP control vector alone (Figures 4B, C).

Next, we tested whether cell specific loss of ASIC1A affected AMPAR/NMDAR ratio in that same neuron, i.e., whether the effects were cell autonomous. We found that AMPAR/NMDAR ratio was significantly increased in Cre-transduced neurons compared to non-transduced or eGFP-transduced neurons (Figures 4D, E). Furthermore, AMPAR/NMDAR ratio in neurons transduced with eGFP alone did not differ from AMPAR/NMDAR ratio in non-transduced neurons. Together, these data suggest that the NAcc is the cellular site of ASIC1A action, and the effects of ASIC1A disruption are cell-autonomous and restricted to neurons in which the channels are disrupted.

### Synaptic changes in dopamine D1 receptor-expressing MSNs

NAcc MSNs are mainly comprised of dopamine D1 eceptor-expressing neurons (D1R^+^-MSNs) and D2 receptor-expressing neurons (D2R^+^-MSNs) (Gerfen, Engber et al., 1990; Kim, Park et al., 2011; Lobo and Nestler, 2011). Differential activation of D1R^+^ and D2R^+^ MSNs have previously been shown to regulate goal-directed and motivated behaviors (Kravitz, Tye et al., 2012; Tai, Lee et al., 2012; Yawata, Yamaguchi et al., 2012). These MSN subtypes differentially exhibit cocaine-induced plasticity (Smith, Lobo et al., 2013; Scofield, Heinsbroek et al., 2016; Zinsmaier, Dong et al., 2022), and D1R^+^ MSNs have been shown to have elevated synaptic responses to cocaine (Lee, Kim et al., 2006; Kim, Park et al., 2011; MacAskill, Cassel et al., 2014). Cell-type specific increase in AMPAR/NMDAR ratio in D1R^+^ neurons in the NAcc is one of these changes reported following cocaine withdrawal (Roberts-Wolfe, Bobadilla et al., 2018).

We wondered if the elevation in AMPAR/NMDAR ratio that we observed in *Asic1a* ^−/−^ mice, occurred in both MSNs subtypes or was cell type-specific. In light of our observation that the effects of ASIC1A disruption were cell autonomous (Figure 4), we hypothesized that increased AMPAR/NMDAR ratio in *Asic1a* ^*−/−*^ mice may be MSN subtype-specific.

**FIGURE 5:**
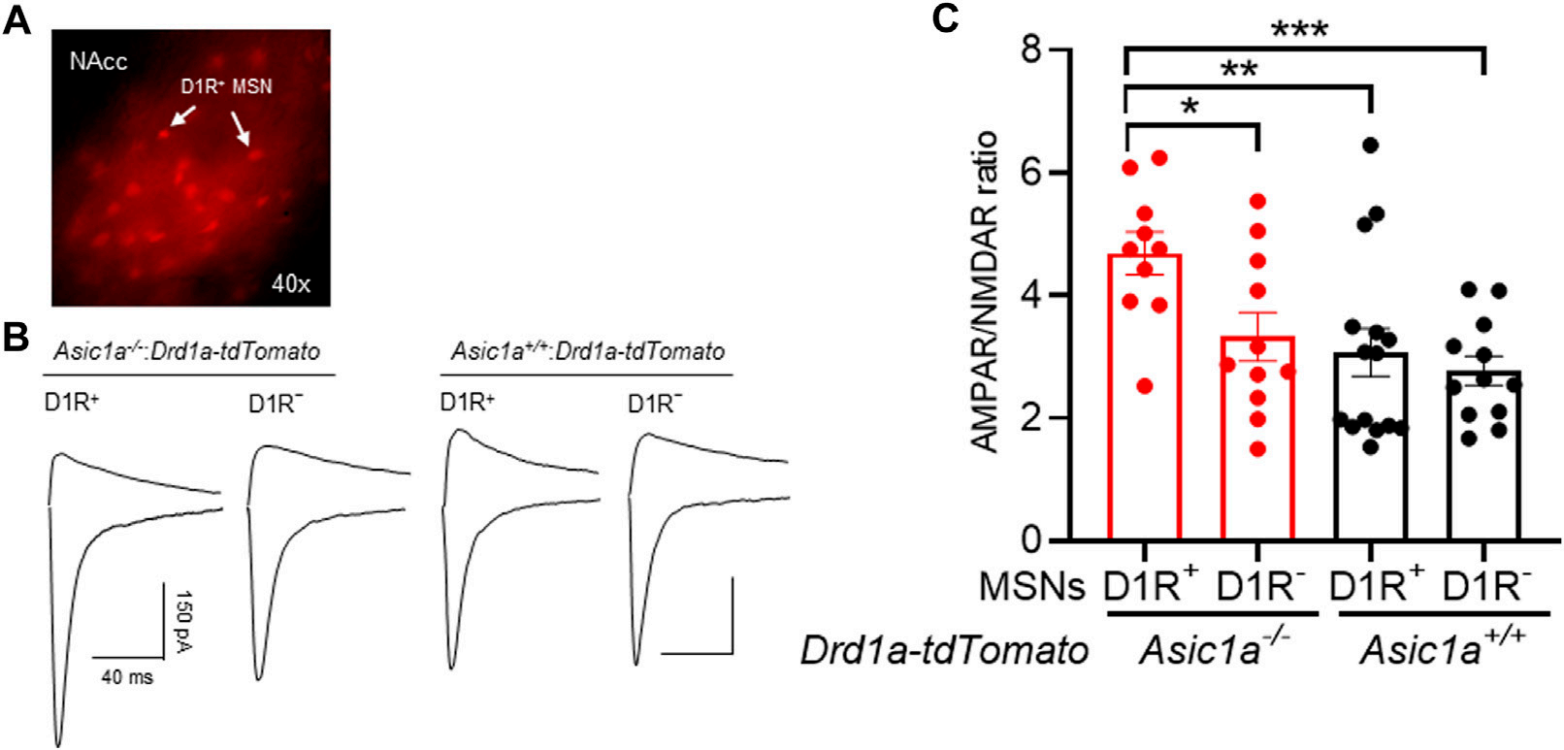
ASIC1A disruption increases the AMPAR/NMDAR ratio preferentially in D1R^+^-MSNs **(A)** Representative image of D1R^+^ neurons (red) in the NAcc under × 40 magnification. **(B)** Representative traces of AMPAR and NMDAR EPSCs **(C)** AMPAR/NMDAR ratio increases in ASIC1A disrupted D1R^+^ MSN not in D1R^−^ MSNs compared to *Asic1a* ^*+/+*^ D1R^+^ and D1R^−^ MSNs. [Interaction F (1, 44) = 2.148 *p* = 0.1499, MSNs subtypes F (1, 44) = 5.341 *p* = 0.0256, genotype F (1, 44) = 8.984 *p* = 0.0045]. Planned contrasts: D1R^+^ MSN vs. D1R^−^ MSNs *Asic1a* ^*−/−*^ *p = 0.0153, *n* = 10–11 neurons from 4 mice; D1R^+^ MSN *Asic1a* ^*−/−*^ vs. D1R^+^ MSN *Asic1a* ^*+/+*^ **p = 0.0026, *n* = 10–15 neurons from 4-5 mice; D1R^+^ MSN *Asic1a* ^*−/−*^ vs. D1R^−^ MSN *Asic1a* ^*+/+*^ ***p = 0.0008, n = 10–12 neurons from 4 mice)*.*

To test this hypothesis, we took advantage of reporter mice expressing tdTomato in D1R^+^ MSNs. We crossed *Drd1a-tdTomato* mice with our *Asic1a* ^*−/−*^ mice to generate *Asic1a* ^*−/−*^:*Drd1a-tdTomato* mice. We prepared acute brain slices from drug naïve *Asic1a* ^*+/+*^:*Drd1a-tdTomato* mice and *Asic1a* ^*−/−*^:*Drd1a-tdTomato* mice and studied AMPAR/NMDAR ratio in NAcc D1R^+^ and non-D1R^+^ (D1R^–^) MSNs. Interestingly, in the *Asic1a* ^*−/−*^ mice we found that AMPAR/NMDAR ratio was significantly increased in tdTomato positive D1R^+^ MSNs, relative to D1R^–^MSNs, and compared to both types of neurons in *Asic1a* ^*+/+*^ mice (Figures 5A–C). In drug-naive *Asic1a* ^*+/+*^ mice the AMPAR/NMDAR ratio in D1R^+^ *versus* D1R^–^MSNs did not significantly differ. Together these data suggest that the cell autonomous effects of ASIC1A disruption may be more pronounced in D1R^+^ MSNs.

### Dependence on protein synthesis

Cocaine withdrawal was previously shown to increase GluA1 protein synthesis in NAcc but not increase protein synthesis in general (Stefanik, Milovanovic et al., 2018), which likely contributes to the increased expression of CP-AMPARs at MSN synapses in withdrawn conditions. Importantly in cocaine-withdrawn rats, the protein synthesis inhibitor anisomycin rapidly reversed the RI to drug-naïve levels and inhibited cocaine seeking, suggesting new GluA1 protein synthesis helps maintain long-lasting changes in RI following cocaine withdrawal, and also promotes cocaine-seeking behavior (Scheyer, Wolf et al., 2014; Werner, Stefanik et al., 2018).

**FIGURE 6:**
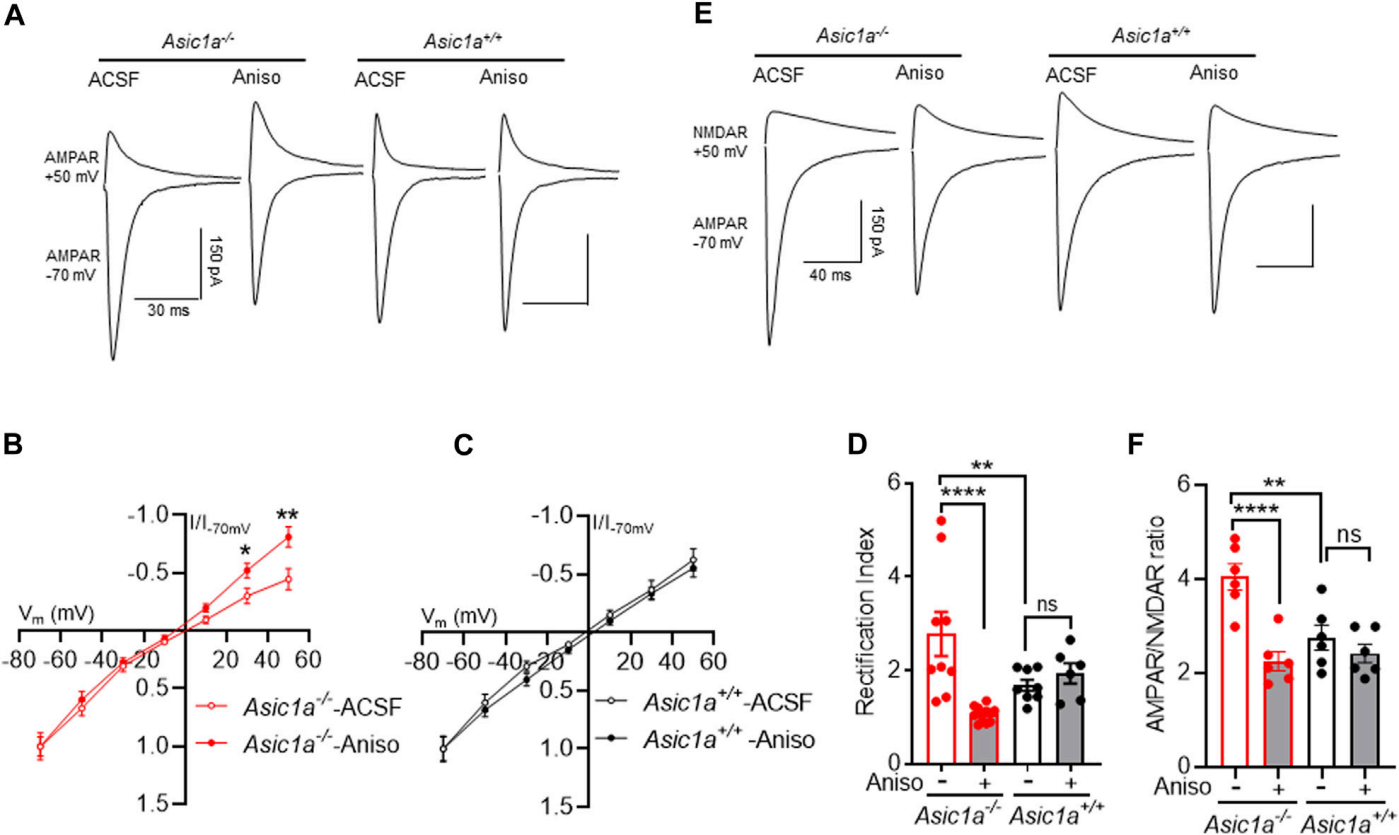
Anisomycin treatment attenuates AMPAR-rectification and AMPAR/NMDAR ratio in *Asic1a* ^*−/−*^ not in *Asic1a* ^*+/+*^ mice **(A)** Traces of artificial cerebrospinal fluid (ACSF) and anisomycin (Aniso) treated AMPAR-mediated EPSCs at −70 mV and +50 mV in NAcc MSNs. **(B)** Anisomycin reverses inwardly rectified I-V curve in *Asic1a* ^*−/−*^ mice. ACSF vs. anisomycin, t (20) = 2.5 *p = 0.0212 (at 30 mV), t (20) = 2.901 **p = 0.0088 (at 50 mV), *n* = 9–13 neurons from 4 mice. **(C)** I-V curve did not change with anisomycin treatment in *Asic1a* ^*+/+*^ mice. ACSF vs. anisomycin, t (12) = 3.099 *p* = 0.7620 (at 30 mV), t (12) = 0.5824 *p* = 0.5711 (at 50 mV), *n* = 6–8 neurons from 3 mice. **(D)** Anisomycin treatment attenuates rectification index in *Asic1a* ^*−/−*^ mice not in *Asic1a* ^*+/+*^ mice [Interaction: F (1, 31) = 13.37 *p* = 0.0009]. Planned contrast; ACSF vs. anisomycin *Asic1a* ^*−/−*^ ****p < 0.0001, *n* = 9–12 neurons from 4 mice; *Asic1a* ^*−/−*^ ACSF vs. *Asic1a* ^*+/+*^ ACSF **p = 0.0063, *Asic1a* ^*+/+*^ ACSF vs. *Asic1a* ^*+/+*^ anisomycin *p* = 0.5466, *n* = 6–8 neurons from 3 mice. **(E)** Traces of AMPAR-mediated EPSCs at −70 mV and NMDAR-mediated EPSCs at +50 mV in NAcc MSNs. **(F)** Anisomycin treatment reverses the AMPAR/NMDAR ratio in *Asic1a* ^*−/−*^ *but not in Asic1a* ^*+/+*^ mice [Interaction: F (1, 20) = 9.380 *p* = 0.0061]. Planned contrast; ACSF vs. anisomycin Asic1a−/− ****p < 0.0001, n = 6 neurons from 3 mice, Asic1a ^*−/−*^ ACSF vs. *Asic1a* ^*+/+*^ ACSF **p = 0.001, Asic1a ^*+/+*^ ACSF vs. *Asic1a* ^*+/+*^ anisomycin *p* = 0.3352, *n* = 6 neurons from 3 mice.

Therefore, we wondered with anisomycin might also reverse the elevated RI and AMPAR/NMDAR ratio in drug-naïve *Asic1a* ^*−/−*^ mice. We pretreated acute brain slices with ACSF or anisomycin (25 µM, 1 h), and subsequently tested RI and AMPAR/NMDAR ratio. Anisomycin strikingly lowered both measures in *Asic1a* ^*−/−*^ mice but not in *Asic1a* ^*+/+*^ mice (Figures 6A–F). Together, these data suggest that the synaptic rearrangements observed in NAcc MSNs were specific to *Asic1a* ^*−/−*^ mice and involved continuous synthesis of GluA1.

## Discussion

Our earlier work suggested that ASIC1A disruption causes changes in multiple measures of synaptic function and structure in NAcc including increases in AMPAR/NMDAR ratio, RI, mEPSC frequency, and dendritic spine density (Kreple, Lu et al., 2014). These changes in drug-naïve *Asic1a* ^*−/−*^ mice resemble changes previously observed in cocaine-withdrawn mice and rats and have been suggested to underlie craving and relapse (Cornish and Kalivas, 2000; Kreple, Lu et al., 2014). The current study identified additional characteristics of *Asic1a* ^*−/−*^ NAcc MSNs that resembled the effects of cocaine withdrawal, including sensitivity to acute cocaine exposure, relative specificity for D1R expressing MSNs, and sensitivity to anisomycin (Table 1).

**TABLE 1:** Similarities in NAcc MSNs between ASIC1A disruption and cocaine withdrawal.

Synaptic parameters	Cocaine withdrawal (mice &/or rats)	Drug naïve *Asic1a* ^*−/−*^ mice
AMPAR/NMDAR ratio compared to drug naïve *Asic1a* ^*+/+*^	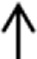 Kourrich, Rothwell et al., (2007); Wolf, (2016); Gupta, Ghobbeh et al., (2022)	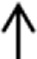 Figure 1 Kreple, Lu et al., (2014)
AMPAR rectification and NASPM sensitivity compared to drug naïve *Asic1a* ^*+/+*^	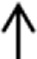 Kourrich, Rothwell et al., (2007); Wolf, (2016); Gupta, Ghobbeh et al., (2022)	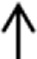 Figure 2 Kreple, Lu et al., (2014)
Dendritic spine density Compared to drug naïve *Asic1a* ^*+/+*^	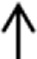 Lee, Kim et al., (2006); Gupta, Ghobbeh et al., (2022)	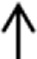 Figure 3 Kreple, Lu et al., (2014)
Normalized by acute cocaine	AMPAR/NMDAR ratio, CP-AMPAR, dendritic spines Boudreau, Reimers et al., (2007); Kourrich, Rothwell et al., (2007); Ceglia, Lee et al., (2017)	Yes (AMPAR/NMDAR ratio, RI, dendritic spine density) Figures 1–3, Kreple, Lu et al., (2014)
Timing of normalization	6–24 h (AMPAR/NMDAR ratio, CP-AMPAR, dendritic spines) Boudreau, Reimers et al., (2007); Kourrich, Rothwell et al. (2007); Ceglia, Lee et al., (2017)	6–4 days (AMPAR/NMDAR ratio), 24 h (RI, dendritic spine density) Figures 1–3
Reversal of Normalization	24 h to 7 days CP-AMPAR Boudreau, Reimers et al., (2007); Kourrich, Rothwell et al., (2007); Ferrario, Li et al., (2010)	4 days–7 days (AMPAR/NMDAR ratio), Figure 1
D1R vs. D2R MSNs	D1R (AMPAR/NMDAR ratio, spine density, CP-AMPAR) MacAskill, Cassel et al., (2014); Zinsmaier, Dong et al., (2022)	D1R (AMPAR/NMDAR ratio) Figure 5
Normalized by anisomycin	Yes (RI) Scheyer, Wolf et al., (2014)	Yes (AMPAR/NMDAR ratio, RI) Figure 6

We further observed that effects of ASIC1A disruption were cell autonomous and did not affect nearby neurons. This observation suggests that synaptic consequences of ASIC1A disruption may be restricted to specific MSNs. Because different MSN subtypes have been suggested to have differing roles in behavior (Lobo and Nestler, 2011), we tested effects of ASIC1A disruption in D1R^+^ MSNs *versus* D1R^–^MSNs and found that ASIC1A disruption resulted increased AMPAR/NMDAR ratio specifically in D1R^+^ MSNs. Previous work suggests that D1R^+^ MSNs promote reward seeking, whereas D2R^+^ MSNs promote aversion (Danjo, Yoshimi et al., 2014; Terrier, Lüscher et al., 2016). Thus, the finding that ASIC1A disruption increases AMPAR/NMDAR ratio in D1R^+^ suggests loss of ASIC1A in these neurons may promote drug-seeking and may account at least in part for the previously observed increase in conditioned place preference to cocaine and morphine in *Asic1a* ^*−/−*^ mice (Kreple, Lu et al., 2014).

We also delineated the timing of the effects of cocaine on synapse function and structure in *Asic1a* ^*−/−*^ mice. Interestingly, a single dose of cocaine reversed the elevated AMPAR/NMDAR ratio, CP-AMPARs, and dendritic spine density in *Asic1a* ^*−/−*^ mice within 24 h. We found no effect of cocaine on AMPAR/NMDAR ratio at 6 h and 7 days post-injection. However, reduced AMPAR/NMDAR ratio was evident at 15 h and persisted until at least 4 days suggesting a specific time window that is similar to the timing of synaptic adaptations previously observed in cocaine-withdrawn mice and/or rats following an injection of cocaine (Boudreau, Reimers et al., 2007; Kourrich, Rothwell et al., 2007; Ferrario, Li et al., 2010) suggesting a similar mechanism of action. Cocaine is rapidly absorbed and metabolized, with peak cocaine levels expected approximately 20 min post-injection (Pettit and Pettit, 1994; Frantz, O'Dell et al., 2007). Thus, the delayed timing of the cocaine effects observed in *Asic1a* ^*−/−*^ mice suggests they are not due to the immediate action on dopamine signaling while cocaine is on board. Rather, they point to a slower mechanism that likely depends on processes such as transcription, translation, and/or post-translational modification. Supporting this observation, inhibiting protein synthesis with anisomycin attenuated both the elevated AMPAR RI and the elevated AMPAR/NMDAR ratio in *Asic1a* ^*−/−*^ mice, but did not alter these measures in *Asic1a* ^*+/+*^ controls. These results suggest that loss of ASIC1A leads to an ongoing synthesis of GluA1 protein or other proteins involved in trafficking CP-AMPARs to NAcc synapses. The mechanisms may thus parallel those suggested to underlie increased CP-AMPARs at synapses following cocaine withdrawal which include increased phosphorylation of GluA1 and increased synthesis of GluA1 protein (Boudreau, Reimers et al., 2007; Scheyer, Wolf et al., 2014; Stefanik, Milovanovic et al., 2018; Werner, Stefanik et al., 2018).

Our observations raise questions about the normal physiological role of ASICs and the mechanisms by which ASIC1A disruption produces synaptic rearrangements. We speculate that ASIC1A plays a homeostatic role in maintaining synaptic stability, and in its absence, synapses are less stable. This state may resemble the synaptic state following cocaine exposure and withdrawal, which has been viewed as immature or hyperplastic (Cornish and Kalivas, 2000; Kourrich, Rothwell et al., 2007; Luscher, 2016; Wolf, 2016). One model put forth to explain effects of cocaine withdrawal focuses on the observed reduction in metabotropic glutamate receptor 1 (mGluR1) signaling and the associated Ca^2+^ release, resulting in an increase in CP-AMPARs being trafficked to synapses (McCutcheon, Loweth et al., 2011a; Loweth, Scheyer et al., 2014a; Loweth, Tseng et al., 2014b). Loss of ASIC1A might similarly reduce synaptic Ca^2+^, as extracellular acidosis induces an increase in intracellular Ca^2+^ that depends on ASIC1A and voltage-gated Ca^2+^ channels (Gupta, Ghobbeh et al., 2022).

Together, these studies increase our understanding of the role of ASIC1A in synaptic physiology and cocaine-induced plasticity. The striking parallels between the synaptic rearrangements induced by cocaine withdrawal and ASIC1A disruption suggest the exciting possibility that potentiating ASIC1A function might protect against insidious synaptic effects of cocaine withdrawal. Consistent with this speculation, our previous results found that potentiating ASIC1A function by disrupting carbonic anhydrase 4 protected against many of the synaptic adaptations induced by cocaine withdrawal and reduced drug seeking in the cocaine withdrawn state (Gupta, Ghobbeh et al., 2022). Similarly, overexpression of ASIC1A in NAcc reduced cocaine self-administration in rats (Kreple, Lu et al., 2014). Additional work will be needed to further delineate these mechanisms, to determine if they extend to other drugs of abuse, and to test if they might be leveraged for therapeutic purposes.

## Data Availability

The original contributions presented in the study are included in the article/Supplementary Material, further inquiries can be directed to the corresponding author.
